# miR-153 inhibits the migration and the tube formation of endothelial cells by blocking the paracrine of angiopoietin 1 in breast cancer cells

**DOI:** 10.1007/s10456-018-9630-9

**Published:** 2018-06-29

**Authors:** Huichun Liang, Fei Ge, Yuhui Xu, Ji Xiao, Zhongmei Zhou, Rong Liu, Ceshi Chen

**Affiliations:** 1 Key Laboratory of Animal Models and Human Disease Mechanisms of Chinese Academy of Sciences & Yunnan Province, Kunming Institute of Zoology, Chinese Academy of Sciences, Kunming, 650223 China; 2 State Key Laboratory of Phytochemistry and Plant Resources in West China, Kunming Institute of Botany, Chinese Academy of Sciences, Kunming, 650201 China; 3 Department of Breast Surgery, The First Affiliated Hospital of Kunming Medical University, Kunming, 650032 Yunnan China

**Keywords:** MiR-153, Angiopoietin 1, Endothelial cell, Tumor angiogenesis, Breast cancer

## Abstract

The sprouting of endothelial cells is the first step of tumor angiogenesis. Our previous study suggests that miR-153 suppresses breast tumor angiogenesis partially through targeting hypoxia-induced factor (HIF1α). In this study, we demonstrated that miR-153 also suppresses the migration and the tube formation of endothelial cells through directly targeting angiopoietin 1 (ANG1) in breast cancer cells. There was a negative correlation between miR-153 and ANG1 levels in breast cancer. miR-153 blocked the expression and secretion of ANG1 in breast cancer cells through binding to *ANG1* mRNA. Conditioned medium from the breast cancer cell, MCF7, treated with miR-153 had no effect on the proliferation of HUVECs, but significantly inhibited the migration and tube formation of HUVECs, which could be rescued by overexpression of ANG1. In addition, miR-153 also directly inhibited the proliferation and migration of MCF7 through downregulation of ANG1. These findings suggest that miR-153 suppresses the activity of tumor cells and the migration and tube formation of endothelial cells by silencing ANG1.

## Introduction

The growth of a solid tumor depends on the blood vessels that provide the tumor with nutrition and oxygen [1]. To provide the necessary components to permit rapid tumor growth, tumor cells activate the endothelial cells of pre-existing blood vessels and promote their sprouting and migration toward the tumor by secreting proangiogenic factors [2–5]. Vascular endothelial growth factor A (VEGFA) and angiopoietin 1 (ANG1) are important proangiogenic factors [3, 6].

ANG1 is a secreted ~ 70-KDa glycoprotein belonging to the angiopoietin family. This family contains three other members: angiopoietin 2 (ANG2), the mouse orthologue angiopoietin 3 (ANG3), and the human orthologue angiopoietin 4 (ANG4). The angiopoietin family contributes to the remodeling and maturation of novel blood vessels by interacting with its tyrosine kinase receptor, Tie2, on the membrane of endothelial cells [7]. ANG2 is the natural antagonist for ANG1 [8, 9] and disrupts angiogenesis by inhibiting the phosphorylation of Tie2 [10].

In addition to stabilizing blood vessels during the late stage of angiogenesis [11–13], ANG1 also promotes the sprouting of endothelial cells at the early stage of angiogenesis through downstream activation of Tie2 via the mitogen-activated protein kinase (MAPK) [14] and phosphoinositide 3-kinase (PI3K) pathways [15]. Because the sprouting of endothelial cells is the first step in angiogenesis [15–18], the growth of a tumor can be inhibited by inactivating ANG1/Tie2 signaling [19–22].

miR-153 is an ancient and conserved miRNA which suppresses the tumor development by downregulating tumor-associated genes, such as *SNAI1* [23], *KLF5* [24], and *MCL-1* [25]. Furthermore, recent work by us and two other groups has shown that stress-responsive miR-153 suppresses tumor angiogenesis by inactivating the HIF1α/VEGFA axis under hypoxic conditions [26–28]. However, the function and the mechanisms of miR-153 activity in tumor angiogenesis are not completely understood, especially under normoxic conditions.

Using an online database (http://www.microrna.org), we found that miR-153 can bind to the three prime untranslated regions (3′UTR) of *ANG1* mRNA. In this study, we demonstrated that miR-153 downregulates the expression of ANG1 in tumor cells and inhibits the migration and tube formation of endothelial cells through blocking the paracrine activity of ANG1.

## Materials and methods

### Cell lines and culture

In this study, we used immortalized breast epithelial cell lines MCF10A and 184B5, and the breast cancer cell lines MDA-MB-231, SUM149PT, Hs578T, HCC1937, HCC1806, MDA-MB-468, MCF7, and T47D. All these cell lines were characterized with short tandem repeat (STR) profiling analysis and were cultured according to the ATCC recommendations. Fetal bovine serum, DMEM, DMEM/F12 (1:1), and 1640 basic media were purchased from Gibco. Primary human umbilical vein endothelial cells (HUVECs) were isolated from neonatal umbilical cord veins and cultured in the EGM-2 Bullet Kit (CC-3162, Lonza) as described in our previous study [27]. All cells were cultured in an incubator with 5% CO_2_ at 37 °C.

### Clinical breast cancer samples and immunohistochemistry

We obtained seven clinical breast cancer tissue samples and their matched normal tissue samples from the First Affiliated Hospital of Kunming Medical University, a procedure that was approved by the human ethics committee of Kunming Institute of Zoology, Chinese Academy of Sciences. These tissues were fixed in 3.7% formalin for 36 h and then treated with paraffin at 65 °C. 4-µm-thick paraffin tissue sections were cut and used in the immunohistochemistry assay. After antigen repairing using a pressure cooker, tissue sections were incubated with the ANG1 primary antibody (23302-1-AP, Proteintech) overnight at 4 °C. The next day we used the anti-mouse/rabbit ultra-sensitive polymer system (PV-8000, ZSGB-BIO, China) to incubate the tissue section. ANG1 expression was detected by using the DAB staining. The images were collected using a microscope.

### Western blot and antibody

We used RIPA lysis buffer [50 mM Tris pH 7.4, 150 mM NaCl, 1% Triton X-100, 1% sodium deoxycholate, 0.1% SDS, and the protease inhibitor cocktail (P8340, Sigma-Aldrich)] to lyse cells and the clinical breast cancer tissue samples by incubating for 30 min on ice. Then, 10–12% SDS–PAGE gels were used to separate proteins. Proteins on the gels were transferred to PVDF membranes (IPVH00010, Millipore). After blocking with 5% skim milk for 1 h at room temperature, the membrane was incubated with primary antibodies against ANG1 (23302-1-AP, Proteintech), GAPDH (60004-1-Ig, Proteintech), and β-ACTIN (60008-1-Ig, Proteintech), and FLAG® M2 (F1804, Sigma-Aldrich) overnight at 4 °C. The next day, the membranes were incubated with secondary anti-rabbit IgG (31460, Thermo Fisher), or anti-mouse IgG antibodies (31430, Thermo Fisher) in 3% skim milk for 40 min at room temperature. Chemiluminescence was detected by using HRP substrate (WBKLS0100, Millipore).

### Real-time PCR

We used TRIzol® (15590-026, Invitrogen) to isolate total RNA of breast cancer tissue and cells. iScript complementary DNA synthesis (170-8891, Bio-Rad) and TaqMan® MicroRNA Reverse Transcription (4366596, Thermo Fisher) kits were used to obtain the cDNA and miR-153, respectively. SYBR Green (4472908, Applied Biosystems) was used to quantify the *ANG1* mRNA and miR-153 in the ABI-7900HT system (Applied Biosystems). The primer sequences of *ANG1* for real-time PCR are as follows: forward, 5′-CAGCGCCGAAGTCCAGAAAAC-3′; and reverse, 5′-CACATGTTCCAGATGTTGAAG-3′. The reverse transcription PCR and the real-time PCR primer sequences for *GAPDH*, 18S rRNA, miR-153, and U6 were described in our previous study [27].

### Dual-luciferase reporter assays

We synthesized complementary DNA oligonucleotides of the predicted binding sequence and mutant sequences of miR-153 on the 3′UTR of *ANG1* mRNA. The predicted binding sequence (WT) was 5′-CCCAAGCTT ACTTTTCCACAATATAGATACGTTTGGATGATTGTTTAATACTAGTCCG-3′. The sequence for a single-point mutation (Mut 1) was 5′-CCCAAGCTT ACTTTTCCACAATATAGATAC***C***TTTGGATGATTGTTTAATACTAGTCCG-3′. The sequence of a three-point mutation (Mut 3) was 5′-CCCAAGCTT ACTTTTCCACAATATA***T***ATA***TA***TTTGGATGATTGTTTAATACTAGTCCG-3′. These oligonucleotides were cloned into the pMIR-Report™ system. After sequencing, the plasmids (800 ng/well) pMIR-ANG1-3′UTR-WT, pMIR-ANG1-3′UTR-Mut1, or pMIR-ANG1-3′UTR-Mut3, together with pCMV-Renilla control (8 ng/well), were transfected into HEK293T cells which had been pre-transfected with miR-153 mimics (50 nM) or negative control (50 nM). At 48 h after transfection, cellular lysate was collected and used to detect luciferase activity (Promega, USA).

### ELISA assay

MCF7, MDA-MB-231, and HCC1937 cells were seeded in 12-well plates. At 8 h after transfection with miR-153 mimics (50 nM), the cellular supernatant was replaced with fresh medium. The conditioned media of the grown breast cancer cell lines were collected into sterilized 1.5-ml tubes 72 h after transfection. After centrifugation for 5 min at 1000 rpm, the supernatant was transferred to a new tube. The secreted levels of ANG1 in the conditioned medium were detected using a Human Angiopoietin-1 Quantikine ELISA Kit (DANG10, R&D systems, Shanghai) according to the manufacturer’s recommendations.

### Ectopic expression of ANG1 in MCF7 cells

We amplified the encoding sequences of the human *ANG1* gene (NM_001146.4) without the miR-153 binding sites, using the cDNA of the HEK293T cell line with the following PCR primers: 5′-CGGGATCCATGACAGTTTTCCTTTCCTT-3′ (forward) and 5′-CCCTCGAGTCAAAAATCTAAAGGTCGAA-3′ (reverse). The ANG1 coding region was cloned into a pCDH-CMV-MCS-EF1-puro-3 × FLAG-3 × HA lentiviral vector which was obtained from Prof. Wen Liu at Xiamen University (Xiamen, Fujian, China). After sequencing, the pCDH-ANG1 expression plasmid and other three packaging plasmids (pMDLg/pRRE, pRSV-Rev, and pCMV-VSV-G) were transfected into the HEK293T cells to prepare lentivirus. At 72 h after transfection, the lentivirus was harvested and used to infect the MCF7 cells. At 48 h later, 2 µg/ml puromycin was added to select ANG1 overexpressing cells.

### Sulforhodamine B (SRB) assays

MCF7, MCF7-pCDH, and MCF7-ANG1 cells were seeded in a 6-cm culture dish and transfected with miR-153 mimics (50 nM) or NC control (50 nM). At 48 h after transfection, the cells were re-seeded in 48-well plates (2 × 10^4^/well), and each treatment group was seeded in six wells. At 8 h after seeding, three of the six wells were fixed in 10% trichloroacetic acid (TCA) for one hour as seeding control wells. At 48 h after seeding, the other three wells were fixed as experimental wells. The cells were stained with 200 µl 0.4% SRB (S9012, Sigma) solution in 1% acetic acid for 10 min. After washing with 1% acetic acid twice, the stain was solubilized with 10 mM unbuffered Tris base (pH 10.5) and the plates were read at 530 nm wavelength. To avoid differences in cell counting, the proliferation of cells was evaluated by using the relative OD (OD_experiment_ − OD_seeding control_).

### EdU assays

Primary HUVECs were seeded on coverslips (BD biosciences) the day before the experiment. The EGM-2 media were discarded the next day. The cells on the cultured slides were washed with 1 × PBS twice. Then the conditioned media derived from the MCF7 cells were used to culture the HUVECs for 6 h. Subsequently, proliferating cells were labeled with EdU for 4 h. The primary HUVECs were then fixed in 3.7% formalin for 20 min at room temperature. DNA synthesis was detected using the Click-iT® EdU Alexa Fluor® 488 Imaging Kit (C10337, Invitrogen) according to the manufacturer’s protocol. Five random visual fields for each treatment group were collected using fluorescence microscopy. Total cells and EdU positive cells were counted using Image-Pro-Plus 6.0 (Media Cybernetics, USA).

### Transwell assays

The migration of MCF7 cells was evaluated using a 24-well format with 8-µm pores (Corning). The bottom side of the membrane of the upper chamber was pre-coated with 600 µl DMEM medium, while the lower chamber was incubated 10% FBS for 2 h. Then, 6 × 10^4^ cells in serum-free medium were added to the upper chamber of each well. Thirty-six hours later, the cells remaining on the superior side of the membrane were removed gently with cotton swab. The cells that had migrated to the bottom side of the membrane were fixed in 3.7% formalin for 20 min at room temperature, followed by staining with 1% crystal violet in 2% ethanol for 30 min. The images were collected, and the number of cells that had migrated were counted using Image-Pro-Plus 6.0.

### Wound healing assays

We used a wound healing assay to investigate the migration of primary HUVECs in conditioned medium. For each well, 3.5 × 10^5^ HUVECs cells were seeded in 12-well plates. The next day, the supernatant was discarded and replaced with conditioned medium derived from MCF7 cells. A 10-µl pipette tip was used to create a wound in the primary HUVECs. At 0 h and at 24 h, images were collected using light microscopy. The wound closure was analyzed with Image-Pro-Plus 6.0.

### Tube formation assays

Matrigel (BD Biosciences)-coated µ-Slide angiogenesis (ibidi GmbH) was used to observed tube formation of primary HUVECs. Then, 1 × 10^4^ primary HUVECs in 50 µl of conditioned medium derived from MCF7 cells were seeded. At 6 h after seeding, images were collected using light microscopy. The total tube length was measured and analyzed with Image-Pro-Plus 6.0.

### Statistical analysis

All data were analyzed by using the SPSS 18.0 (SPSS Inc., USA) and were shown as the mean ± standard deviation. Each experiment was repeated at least three times. The differences between two groups were analyzed by two-tailed *t* test, and *p* values smaller than 0.5 were considered to be statistically significant.

## Results

### ANG1 is highly expressed in clinical breast cancer tissues

**Fig. 1:**
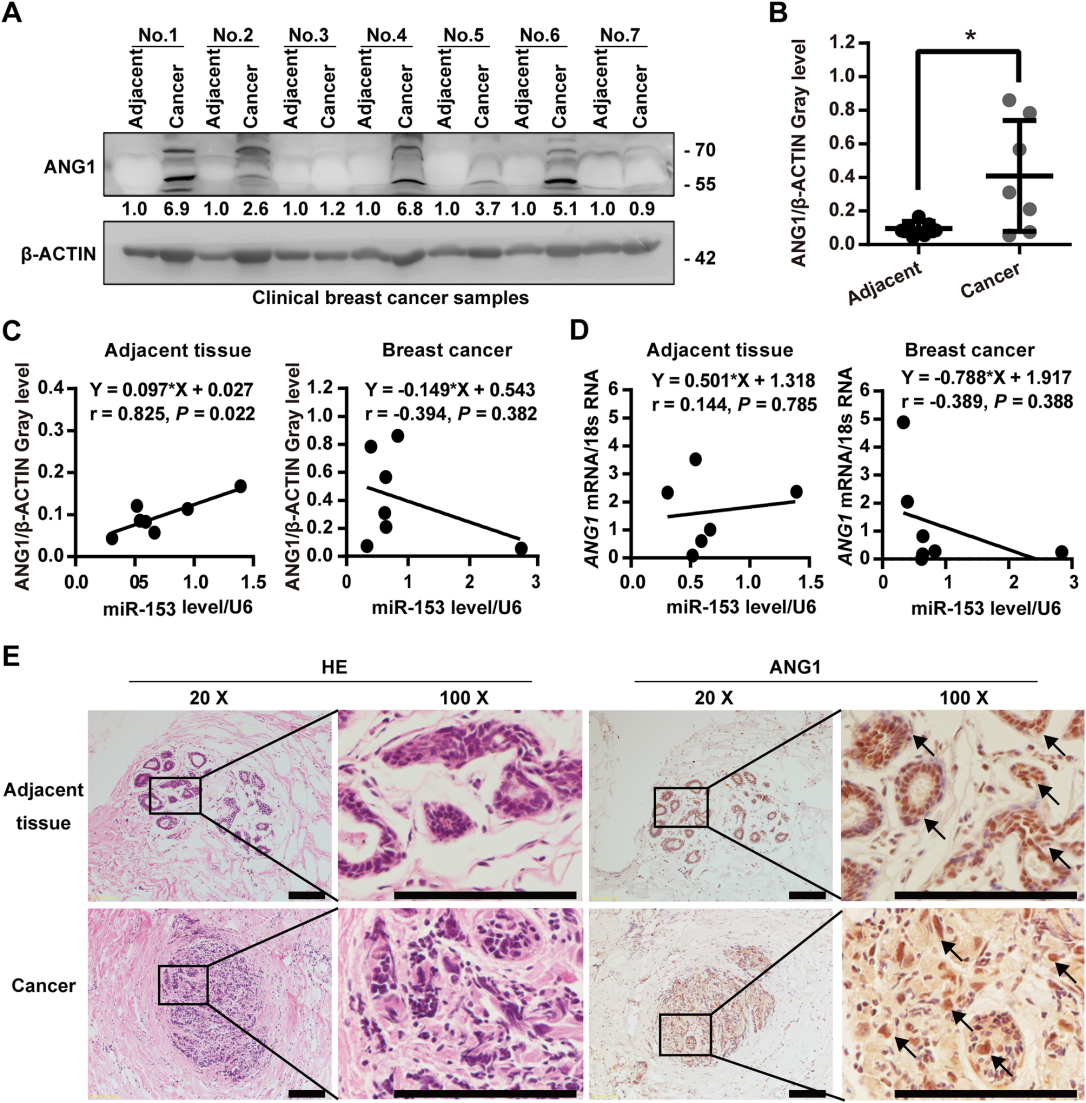
ANG1 is highly expressed in clinical breast cancer tissues. **a** Protein levels of ANG1 in breast cancer and matched adjacent tissues were detected by Western blot. **b** The quantitative results of **a**. *p < 0.05, *t* test. **c** The correlative analysis of miR-153 and ANG1 protein levels in breast cancer and matched adjacent tissues. There is a negative correlative trend between miR-153 and ANG1 at protein level in seven breast cancer tissues. **d** The correlation analysis of miR-153 and *ANG1* mRNA levels in breast cancer and matched adjacent tissues. There is a negative correlative trend between miR-153 and *ANG1* mRNA in seven breast cancer tissues. **e** Immunohistochemical staining of ANG1 in breast cancer and matched adjacent tissues. Representative images are shown. Scale bar, 100 µm

To determine ANG1 expression levels and the correlation between ANG1 and miR-153 levels in breast cancer, we collected seven patient samples which included breast cancer tissues and matched adjacent normal tissues. Although some breast cancer tissues showed no expression of ANG1, overall the ANG1 protein levels in the breast cancer tissues were significantly higher than those in the adjacent tissues as shown by Western blot (Fig. 1a, b). Immunohistochemistry results indicated that ANG1 was largely expressed in the normal breast ductal epithelial cells, but its expression was increased in breast cancer cells (Fig. 1c). We further detected the expression of miR-153 and *ANG1* mRNA in these sample tissues using RT-qPCR, and we found that there was a consistent negative correlative trend between miR-153 and ANG1 at the protein and at the mRNA levels in breast cancer tissues (Fig. 1d, e).

### miR-153 suppresses the ANG1 expression and secretion in breast cancer cells

**Fig. 2:**
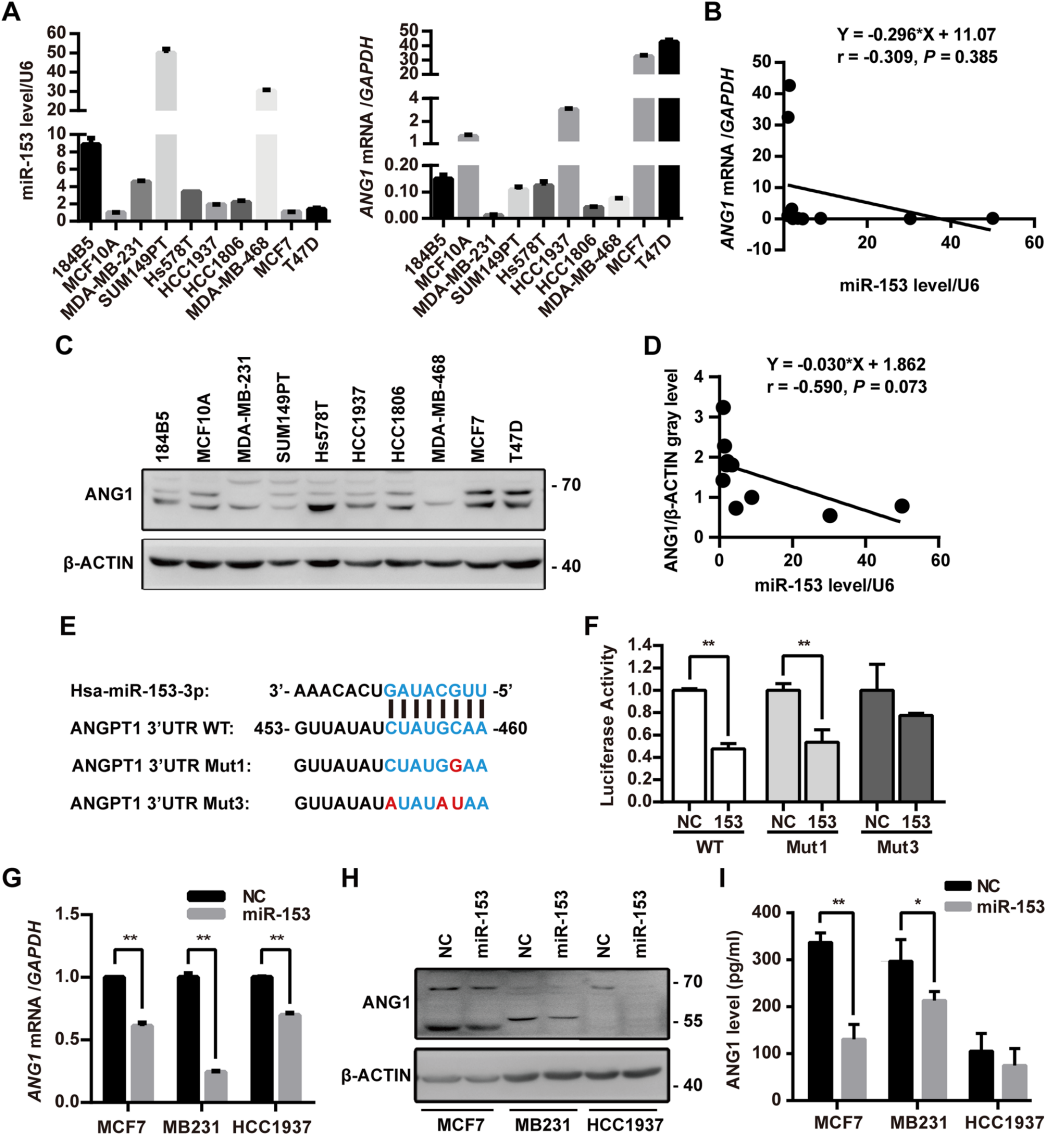
miR-153 suppresses the expression and secretion of ANG1 in breast cancer. **a** Expression levels of miR-153 and *ANG1* mRNA in the breast cancer cell lines was detected by RT-qPCR. **b** The correlative analysis of miR-153 and *ANG1* mRNA in **a. c** Protein levels of ANG1 in breast cancer cell lines were examined by Western blot. **d** The correlation analysis of miR-153 and ANG1 protein levels for **c. e** The predicted binding sequence and the mutant sequences of miR-153 at the 3′UTR of *ANG1* mRNA. **f** miR-153 mimics (50 nM) significantly inhibited the luciferase activity of the pMIR-*ANG1*-3′UTR-WT but not the pMIR-*ANG1*-3′UTR-Mut3 in the HEK293T cell line. **p < 0.01, *t* test. **g** miR-153 mimics (50 nM) downregulated the mRNA levels of ANG1 in three breast cancer cell lines (MCF7, MDA-MB-231, and HCC1937). **p < 0.01, *t* test. **h** miR-153 mimics (50 nM) downregulated protein levels of ANG1 including the non-glycosylated (~ 55 kDa) and the glycosylated (~ 70 kDa) formats in the MCF7, MDA-MB-231, and HCC1937 cell lines. **i** miR-153 mimics (50 nM) significantly decreased the secretion of ANG1 in MCF7 and MDA-MB-231 cell lines. *p < 0.05, **p < 0.01, *t* test

Among the breast cancer cell lines, the expression of ANG1 and miR-153 showed a negative correlation at both the mRNA (Fig. 2a, b) and protein levels (Fig. 2c, d). To test whether miR-153 directly targets ANG1, we sub-cloned the predicted binding sequence and mutant sequences derived from *ANG1* by using the pMIR-Report™ system and performed a luciferase assay. In HEK293T cells, miR-153 mimics significantly inhibited the luciferase activities of the plasmids with the wildtype sequence or the single-point mutation but not the three-point mutation (Fig. 2e, f). We additionally transfected the miR-153 mimics into three breast cancer cell lines (MCF7, MDA-MB-231, and HCC1937), and we found that miR-153 mimics downregulated the expression of ANG1 and decreased the secretion of ANG1 (Fig. 2g–i).

### miR-153 inhibits MCF7 proliferation and migration by silencing ANG1

**Fig. 3:**
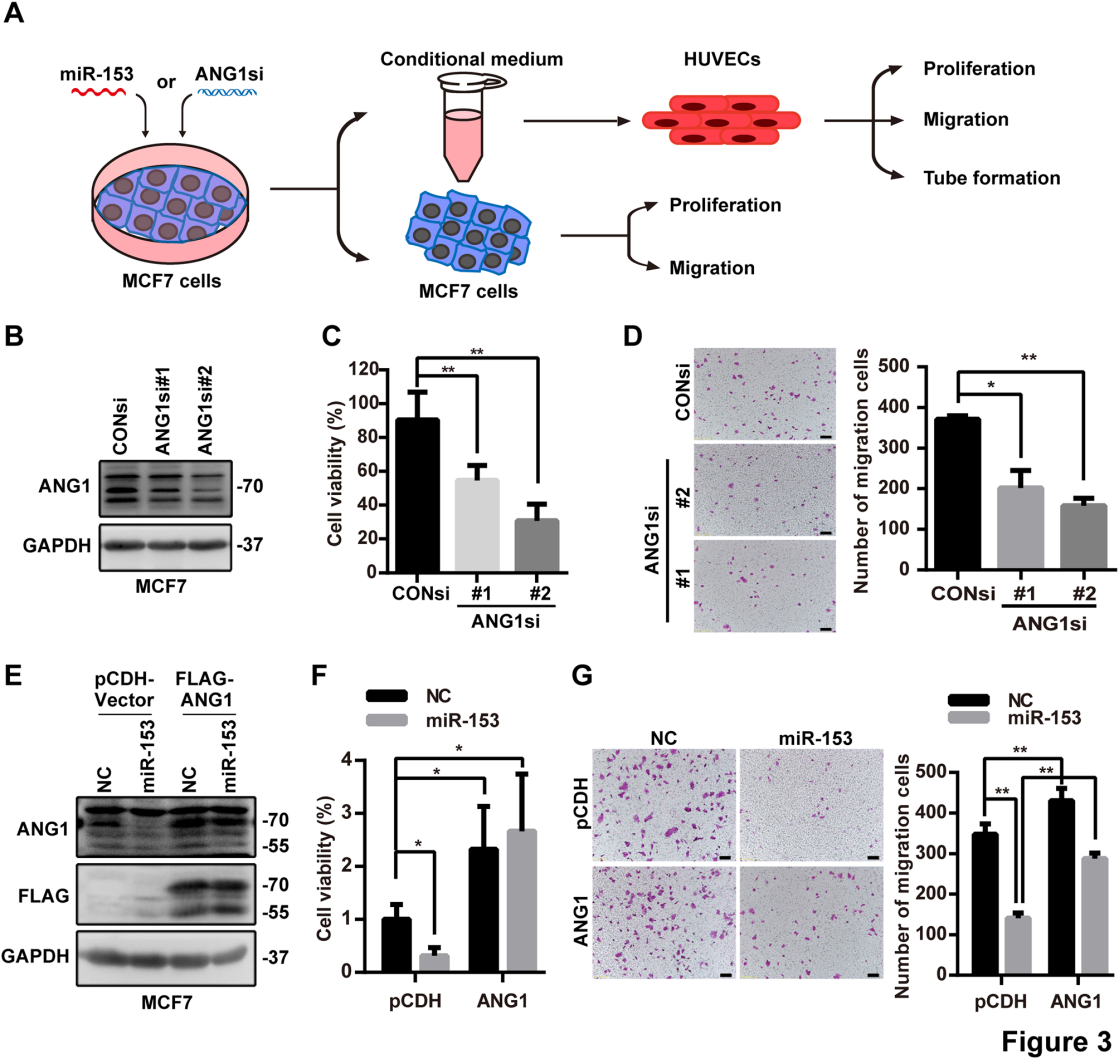
miR-153 suppresses MCF7 proliferation and migration by silencing ANG1. **a** A schematic illustration of cell treatment, collection and detection. **b** Downregulation of ANG1 protein levels after ANG1 siRNA (50 nM) treatment for 72 h in MCF7 cells as detected by Western blot. **c** Knockdown of ANG1 by siRNA decreased cellular viability of MCF7 cells as characterized by the SRB assay. **p < 0.01, *t* test. **d** Knockdown of ANG1 with siRNA inhibited the migration of MCF7 cells characterized with the transwell assy. The representative images are shown, and the scale bar is 100 µm. *p < 0.05, **p < 0.01, *t* test. **e** Ectopic expression of ANG1 in MCF7 without the miR-153 binding site. **f** Ectopic expression of ANG1 increased cellular viability of MCF7 cells and rescued the inhibition of miR-153, shown with the SRB assay. *p < 0.05, *t* test. **g** Ectopic expression of ANG1 increased the migration of MCF7 cells and rescued the inhibition of miR-153, shown using the transwell assay. Representative images are shown, and the scale bar is 100 µm. **p < 0.01, *t* test

We transfected the MCF7 cells with ANG1 siRNAs, and we performed the SRB and Transwell assays to evaluate the function of ANG1 in MCF7. Knockdown of ANG1 decreased the proliferation and migration of MCF7 cells (Fig. 3a, b). To evaluate whether miR-153 inhibits the proliferation and migration of MCF7 cells through ANG1, we stably overexpressed ANG1 in MCF7 (Fig. 3e) and found that the ANG1 overexpression rescued proliferation and migration and protected against miR-153-mediated inhibition. These findings suggest that miR-153 suppresses the proliferation and migration of MCF7 cells through silencing ANG1.

### miR-153 suppresses the migration and tube formation of primary HUVECs through blocking the paracrine of ANG1 in MCF7 cells

**Fig. 4:**
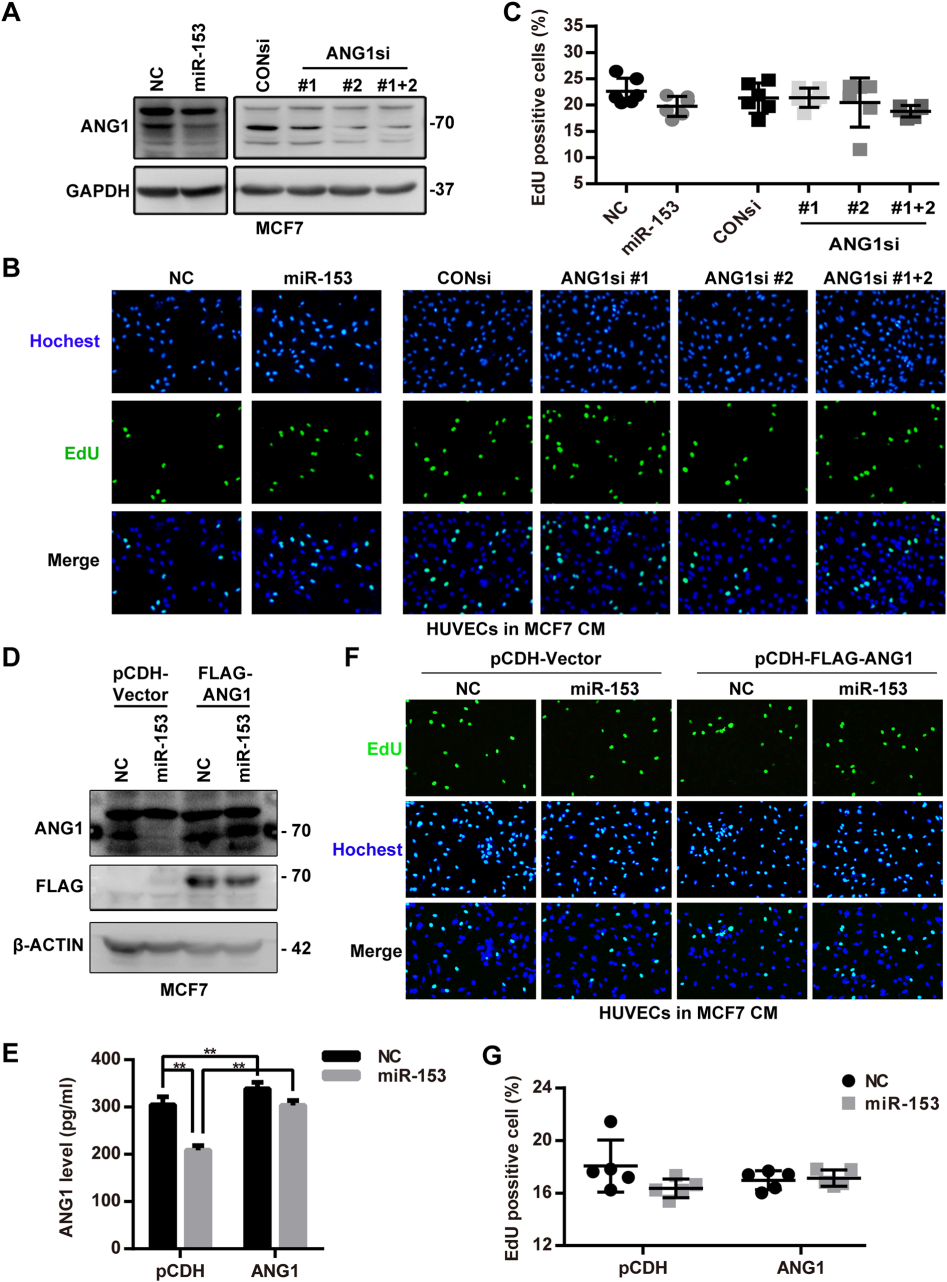
ANG1 from MCF7-conditioned media has no influence on HUVEC proliferation. **a** Downregulation of ANG1 protein levels after miR-153 and ANG1 siRNAs treatment. **b** MCF7-conditioned media from cells treated with the miR-153 or the ANG1 siRNAs had no effect on the proliferation of the primary HUVECs, characterized with the EdU assay. Representative images are shown. “CM” means the conditioned medium. **c** The quantitative results of **b. d** Ectopic expression of ANG1 without the binding site of miR-153 in MCF7 cells. **e** Ectopic ANG1 expression in MCF7 cells was detected using the ELISA assay. **p < 0.01, *t* test. **f** MCF7-conditioned media with ectopic ANG1 had no influence on the proliferation of primary HUVECs as characterized by EdU assay. Representative images are shown. **g** The quantitative results of **f**

**Fig. 5:**
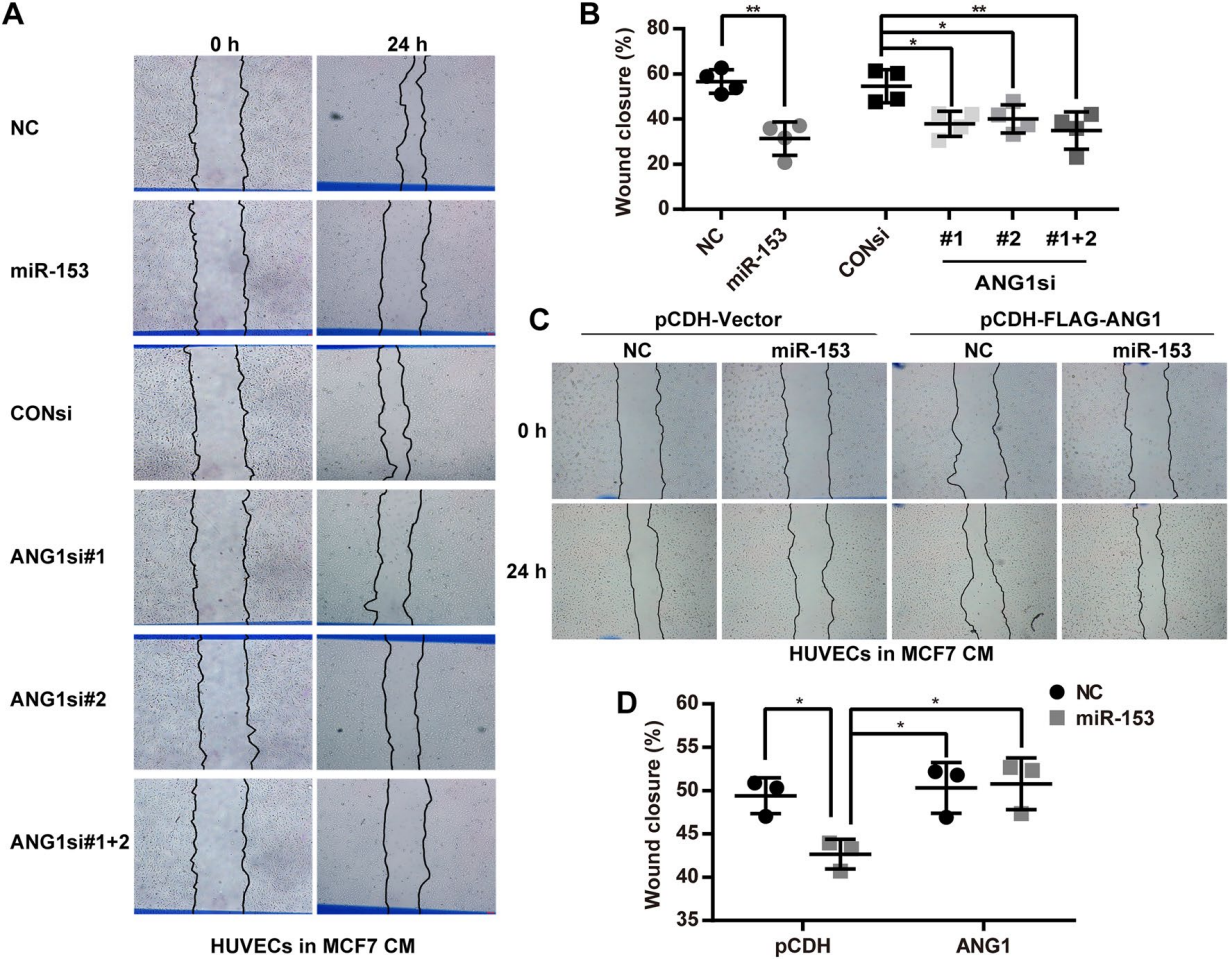
miR-153 inhibits the migration of primary HUVECs through suppression of the secretion of ANG1 from MCF7 cells. **a** MCF7-conditioned media from cells treated with the miR-153 or the ANG1 siRNAs inhibited the migration of the primary HUVECs in a wound healing assay. Representative images are shown. The “CM” means the conditioned medium. **b** The quantitative results of **a**. *p < 0.05, **p < 0.01, *t* test. **c** The MCF7-conditioned medium with the ectopic ANG1 expression increased the migration of the primary HUVECs and rescued the inhibition of miR-153 by the wound healing assay. The representative images are shown. **d** The quantitative results of **c**. *p < 0.05, *t* test

**Fig. 6:**
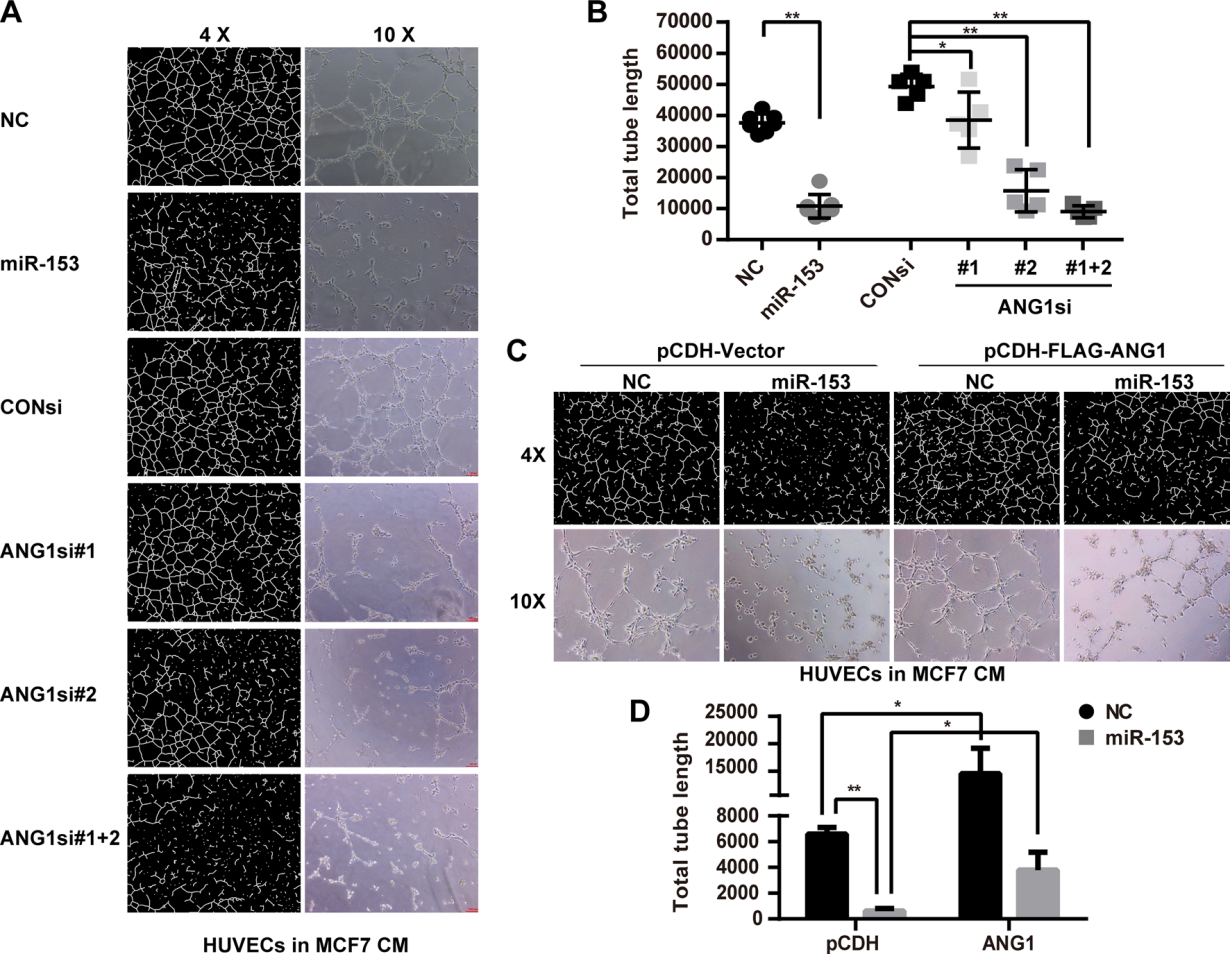
miR-153 inhibits the tube formation of the primary HUVECs through suppressing the secretion of ANG1 from MCF7 cells. **a** MCF7-conditioned medium with the miR-153 or the ANG1 siRNAs treatment inhibited the tube formation of the primary HUVECs by the tube formation assay. The representative images are shown. The “CM” means the conditioned medium. **b** The quantitative results of **a**. *p < 0.05, **p < 0.01, *t* test. **c** MCF7-conditioned medium from cells treated with the ectopic ANG1 increased the tube formation of the primary HUVECs and rescued the inhibition of miR-153 as characterized with a tube formation assay. Representative images are shown. **d** The quantitative results of **c**. *p < 0.05, **p < 0.01, *t* test

We transfected miR-153 mimics or ANG1 siRNAs into MCF7 cells with or without the ectopic ANG1 expression and harvested the conditioned media from the MCF7 cells to incubate with primary HUVECs. The EdU assay suggested that the conditioned media of MCF7 cells incubated with the miR-153 or the ANG1 siRNAs treatment did not affect the proliferation of primary HUVECs (Fig. 4b, c). Additionally, conditioned media of MCF7 cells with ectopic ANG1 expression also did not increase the proliferation of the primary HUVECs (Fig. 4f, g). However, conditioned media from MCF7 cells treated with miR-153 or ANG1 siRNAs decreased the migration and the tube formation of primary HUVECs (Figs. 5a, b and 6a, b), and inhibition of miR-153 could be rescued by the ectopic ANG1 expression (Figs. 5c, d and 6c, d). These findings indicate that miR-153 inhibits primary HUVECs migration and tube formation through suppression of the secretion of ANG1 from MCF7 breast cancer cells.

## Discussion

In this study, we demonstrated that miR-153 inhibits the migration and tube formation of endothelial cells through silencing the expression of ANG1 in breast cancer cells. First, there was a negative correlative trend between miR-153 and ANG1 in both the clinical breast cancer samples and the breast cancer cell lines. Second, miR-153 downregulated the expression of ANG1 in the MCF7, the MDA-MB-231, and the HCC1937 breast cancer cell lines by binding to the 3′UTR of *ANG1* mRNA. Importantly, miR-153 inhibited HUVEC migration and tube formation, but not proliferation. Finally, miR-153-mediated inhibition of HUVEC migration and tube formation was rescued by overexpression of ANG1 in MCF7 cells.

Endothelial cell sprouting is the initial step in angiogenesis [29]. We observed that miR-153 suppressed the ability of primary HUVECs to migrate and to form tubes through blocking the paracrine activity of ANG1 in breast cancer cells. The ability of ANG1 to mediate the sprouting of endothelial cells has been reported in previous studies. The mechanisms by which ANG1 promotes the sprouting of endothelial cells may be associated with the activation of the PI3K and PKCζ/β-catenin/Rac1 pathways after Tie2 phosphorylation [15, 17]. The PI3K inhibitors, the PKCζ siRNAs, and the ANG1/Tie2 antagonist ANG2 inhibit endothelial cell migration and sprouting [10, 15, 17].

In this study, we showed that conditioned media of MCF7 cells treated with miR-153 or the ANG1 siRNAs did not inhibit HUVEC proliferation. Furthermore, conditioned media with ANG1 overexpression did not promote proliferation of HUVECs. In contrast to our results, exogenous ANG1 protein has been previously reported to increase endothelial cell proliferation [14, 18]. In these previous studies, exogenous ANG1 showed weak mitogenic activity only when its concentration was more than 20 ng/ml. In this study, the concentrations of ANG1 in the conditioned medium of MCF7 cells were between 0.1 ng/ml and 0.4 ng/ml. It is reasonable that the low concentration of ANG1 in the conditioned medium derived from MCF7 cells had no effect on the proliferation of HUVECs. These results suggest that at the early stage of tumor angiogenesis, tumor cells-secretion of ANG1 mainly affects endothelial cell sprouting and migration but not proliferation.

In a small number of clinical breast cancer samples, we found that expression levels of ANG1 in cancer tissues were significantly higher than those in the adjacent normal tissues. It has been reported that the removal of the BRCA1/CtIP/ZBRK1 repressor complex on the *ANG1* promotor causes high expression of ANG1 in tumor cells, and promotes tumor growth by accelerating angiogenesis [19]. In addition to promoting the endothelial cell’s migration and tube formation, ANG1 overexpression also promoted MCF7 breast cancer cell’s proliferation and migration. To date, the functions of ANG1 in tumor cells have not been reported. These mechanisms deserve further investigation.

Our previous study and two other studies conducted at the same time suggest that miR-153 inhibits endothelial cell activity and tumor angiogenesis through downregulating the hypoxia-induced HIF1α/VEGFA pathway [27, 28, 30]. In this study, miR-153 inhibited the migration and tube formation of endothelial cells through blocking the paracrine activity of ANG1 in MCF7 breast cancer cells under normoxia. In tumor angiogenesis, especially at the early stage, the VEGFA and the ANG1/Tie2 pathways play a synergistic role [18, 20, 21, 31, 32]. When VEGFA is blocked, the ANG1/Tie2 pathway is thought to be the major contributor to tumor angiogenesis and tumor growth [22]. miR-153 can inhibit both the HIF1α/VEGFA and ANG1/Tie2 pathways to suppress tumor growth and angiogenesis.

In summary, our work reveals that miR-153 inhibited the migration and tube formation of primary HUVECs through directly inhibiting the expression and secretion of ANG1 in MCF7 breast cancer cells. Furthermore, miR-153 suppressed the proliferation and migration of MCF7 by silencing ANG1. Our findings provide a new perspective for clarifying the mechanisms of miR-153 in tumor angiogenesis, and suggest that miR-153 can be developed as a powerful small molecule inhibitor for tumor angiogenesis therapeutics.
